# Experimental observations of the effects of intermolecular Van der Waals force on entropy

**DOI:** 10.1038/s41598-022-11093-z

**Published:** 2022-05-02

**Authors:** Matthew David Marko

**Affiliations:** Marko Motors LLC, Seaside Heights, NJ USA

**Keywords:** Thermodynamics, Fluid dynamics

## Abstract

An experimental effort was conducted to measure the change in internal energy of non-ideal carbon dioxide as its volume rapidly expanded with the sudden opening of a valve from one to two compressed gas cylinders. This was achieved by measuring the mass heat capacity of the gas cylinders and the manifold-valve, and measuring the change in temperature from the sudden doubling of volume of the non-ideal carbon dioxide. It was determined that an empirical equation for the change in internal energy of a non-ideal fluid was more accurate than previous methods used for estimating the change in internal energy by estimating the change in entropy. With this empirical equation, a theoretical ideal Stirling cycle heat engine that exceeds the Carnot efficiency was realized by utilizing non-ideal carbon dioxide as a working fluid.

## Introduction

In an earlier publication^1^, a theoretical Stirling cycle heat engine utilizing a real working fluid with significant intermolecular attractive (and repulsive) Van der Waals force^2–22^ was proposed. The intermolecular attractive Van der Waals force would both decrease the required work input during the cold isothermal compression, as well as reduce the work output recovered during the hot isothermal expansion. If one were to look at empirical equations of state for real fluids, such as Redlich–Kwong^23^ and Peng–Robinson^24^, it becomes clear that the intermolecular attractive force increases with decreasing temperatures. Because of the temperature dependence of the Van der Waals force, which increase in strength with decreasing temperature, the reduction in cold work input is greater than the loss of the hot work output; therefore, the ideal efficiency of this macroscopic heat engine could in theory exceed the Carnot efficiency ${\eta }_C$

1 $$\begin{aligned} {\eta }_C & = {} {1-{\frac{T_L}{T_H}}}. \end{aligned}$$

Originally there were measurements of the enthalpy of vaporization of water^25,26^; when calculating for the change in internal energy during vaporization, which is isothermal expansion of a real fluid, it was observed that the change in specific internal energy ${{\delta }u}_{{\delta }T=0}$ (J/kg) during isothermal compression and expansion followed a distinct empirical equation

2 $$\begin{aligned} {{\delta }u}_{{\delta }T=0} & = {} {{{a'}{\cdot }{{T}^{-0.25}}}{\cdot }{({\rho _1}-{\rho _2})}}, \nonumber \\ {a'} &= {} {\frac{0.21836}{9{\cdot }{({2^{\frac{1}{3}}-1})}}}{\cdot }{\frac{R_{G}^{2}{\cdot }{T_{C}^{2.5}}}{{P_{C}}}}. \end{aligned}$$

where ${\rho }_1$ and ${\rho }_2$ (m$^3$/kg) represent the density, *T* (K) represents the absolute temperature, $R_G$ (J/kg K) represents the gas constant, $T_C$ (K) represents the critical temperature, and $P_C$ (Pa) represents the critical pressure. This equation has been found to match well for numerous fluids^1,25–50^.

For non-isothermal changes in specific internal energy of a fluid ${{\delta }u}$ (J/kg), one must include the specific intermolecular kinetic energy $u_{KE}={C_V}{\cdot }{R_G}{\cdot }{T}$ (J/kg)^51^,

3 $$\begin{aligned} {{\delta }u} &= {} {{C_V}{\cdot }{R_G}{\cdot }{{\delta }{T}}}-{{{a'}{\cdot }{{T}^{-0.25}}}{\cdot }{{\delta }{\rho }}}, \nonumber \\ {a'}&= {} {\frac{0.21836}{9{\cdot }{({2^{\frac{1}{3}}-1})}}}{\cdot }{\frac{R_{G}^{2}{\cdot }{T_{C}^{2.5}}}{{P_{C}}}}. \end{aligned}$$

where *T* (K) represents the absolute temperature, $R_G$ (J/kg K) represents the gas constant, and ${C_V}$ is equal to the number of degrees of freedom of the molecule plus one half (ex. monatomic fluids $C_V=1.5$, diatomic fluids $C_V=2.5$, etc). By integrating Eq. (3) from a given density $\rho$ (kg/m$^3$) to infinitely low density (a true ideal gas) to find the intermolecular potential energy, and the temperature from absolute zero to the current temperature *T*, one can calculate the total specific internal energy *u* (J/kg) with Eq. (4)

4 $$\begin{aligned} {u} &= {} {{C_V}{\cdot }{R_G}{\cdot }T}-{{{a'}{\cdot }{\rho }}{\cdot }{{T}^{-0.25}}},\nonumber \\ {a'} &= {} {\frac{0.21836}{9{\cdot }{({2^{\frac{1}{3}}-1})}}}{\cdot }{\frac{{R_G^2}{\cdot }{T_C^{2.5}}}{{P_C}}}. \end{aligned}$$

Clausius’ Theorem for the second law^2^

5 $$\begin{aligned} {{\oint }{\frac{{\delta }q}{T}}}\le & {} 0, \end{aligned}$$

states that any internally reversible thermodynamic cycle must generate a positive entropy ${\delta }s_u{\ge }0$ to the surrounding universe, where the change in specific entropy ${\delta }s$ (J/kg K) is defined as^3–7^

6 $$\begin{aligned} {\delta }{s} &= {} \frac{{\delta }q}{T}, \end{aligned}$$

where *T* (K) is the absolute temperature, and ${\delta }q$ (J/kg) represent the heat transferred per unit mass. If a fluid were to consistently follow Eq. (5), then the change in specific internal energy ${{\delta }u}$ (J/kg) would consistently follow Eq. (7)^1,3,4,6^

7 $$\begin{aligned} {\delta }{u} &= {} {{C_V}{\cdot }{R_G}{\cdot }{{\delta }T}}+{\left \{{T{\cdot }{(\frac{{\partial }P}{{\partial }T}})}_V-{P} \right \}{\cdot }{{\delta }v}}. \end{aligned}$$

It should be noted that in most of the experimental measurements of the enthalpy of vaporization^1,25–50^ there is great similarity between Eqs. (3) and (7).

Clausius’ Eq. (5) makes intuitive sense for a reversible thermodynamic process utilizing an ideal-gas as its working fluid. With a real-fluid, however, intermolecular Van-der-Waals force impact the molecular behavior and thermodynamic properties. In most published references and tables, the specific internal energy *u* (J/kg) is often set to zero at an arbitrary point (often the triple-point), and calculated assuming Eq. (7), which was formulated for the purpose of holding Eq. (5) applicable.

In contrast, Eq. (3) is an empirical equation based on measurements of the change in internal energy for numerous different molecules during vaporization. In addition, Eq. (3) makes physical sense, as it includes both the internal kinetic energy of the molecules as defined with the Kinetic Gas Theory^51^, as well as the intermolecular potential energy. Lennard-Jones^6,52,53^, a well-established approximation for the potential energy from intermolecular attractive and repulsive Van der Waals force, assumes the attractive force is inverse proportional to the molecular distance to the sixth power $r^{-6}$ (m$^{-6}$); the attractive intermolecular force is thus inverse proportion to the specific volume squared $v^{-2}$ (m$^{-6}$), and this is observed in most empirical equations of state for a real fluid^23,24^. Integrating a potential force inverse-proportional to the specific volume squared would yield a potential energy inverse proportional to the specific volume *v* (m$^3$/kg), or proportional to the density $\rho$ (kg/m$^3$), as observed in Eqs. (3) and (4).

$$\begin{aligned} {{\int }_{v}^{\infty }}{\frac{{\delta }v_0}{v_0^2}}={\frac{1}{v}}={\rho }. \end{aligned}$$

## Non-ideal stirling cycle heat engine

A Stirling cycle heat engine^1,4^ has isothermal (constant temperature) compression at a cold sink (Stage 1–2), isochoric (constant volume) heating to a hot temperature (Stage 2–3), isothermal expansion at the hot temperature source (Stage 3–4), and isochoric cooling back to the cold temperature (Stage 4–1). For a true, ideal-gas Stirling engine to reach the Carnot efficiency (Eq. 1), it is necessary for all of the heat output from the isochoric cooling to go to the isochoric heating.

As a demonstration, carbon dioxide will be the working fluid, with a cold temperature of 32 $^{\circ }$C and a hot temperature of 82 $^{\circ }$C. The density will shift from 70 to 700 kg/m$^3$. Using Eq. (1), the Carnot efficiency ${\eta }_C$

$$\begin{aligned} {\eta }_C={1-{\frac{32+273.15}{82+273.15}}}=14.08\%. \end{aligned}$$

The thermodynamic properties of this Stirling cycle engine are tabulated in Table 1, using both Eq. (4) for the specific internal energy *u* (J/kg) and the National Institute of Standards and Technology (NIST) Chemistry WebBook ^54^. The pressures used to calculate the specific work inputs and outputs $w={\int }P{\cdot }{\delta }v$ (J/kg) from NIST^54^ are tabulated in Table 2. The efficiency is calculated as

8 $$\begin{aligned} {\eta } & = {} -{\frac{{w_{12}}+{w_{34}}}{{q_{23}}+{q_{34}}+{q_{41}}}}. \end{aligned}$$

**Table 1:** The stages of the non-ideal Stirling cycle heat engine, as well as the specific work inputs and outputs *w* (J/kg), specific heat inputs and outputs *q* (J/kg), and specific internal energies *u* (J/kg) from both *NIST*^54^ and calculated *calc* with Eq. (4).

Stage	P (Pa)	T (K)	$\rho$ (kg/m$^3$)	$u_{NIST}$ (J/kg)	$u_{calc}$ (J/kg)
1	3,334,500	305.15	70	430,810	189,575
2	8,650,400	305.15	700	275,020	79,771
3	26,745,000	355.15	700	322,020	117,374
4	4,132,200	355.15	70	468,660	223,091
Stages	w (J/kg)	${\delta }u_{NIST}$ (J/kg)	$q_{NIST}$ (J/kg)	${\delta }u_{calc}$ (J/kg)	$q_{calc}$ (J/kg)
12	69,149	$-155,790$	$-224,939$	$-109,804$	$-178,953$
23	0	47,000	47,000	37,603	37,603
34	$-105,515$	146,640	252,155	105,717	211,232
41	0	$-37,850$	$-37,850$	$-33,516$	$-33,516$

**Table 2:** The pressures and specific internal energies versus the density $\rho$, taken from NIST^54^, and used to solve the work and heat inputs and outputs listed in Table 1. The pressure $P_L$ (MPa) and specific internal energy $u_L$ (kJ/kg) is at 32 $^{\circ }$C, and the pressure $P_H$ (MPa) and specific internal energy $u_H$ (kJ/kg) is at 82 $^{\circ }$C.

$\rho$ (kg/m$^3$)	$P_{L}$ (MPa)	$P_{H}$ (MPa)	$u_{L}$ (kJ/kg)	$u_{H}$ (kJ/kg)
70	3.3345	4.1322	430.81	468.66
88.146	3.5471	5.0367	428.9	463.45
107.63	3.7598	5.9412	426.92	457.93
128.64	3.9724	6.8457	424.86	452.08
151.42	4.185	7.7502	422.73	445.85
176.22	4.3977	8.6548	420.5	439.22
203.31	4.6103	9.5593	418.18	432.14
232.93	4.823	10.464	415.74	424.59
265.24	5.0356	11.368	413.18	416.58
300.17	5.2482	12.273	410.47	408.18
337.28	5.4609	13.177	407.6	399.53
375.61	5.6735	14.082	404.52	390.85
413.83	5.8861	14.986	401.2	382.42
450.56	6.0988	15.891	397.59	374.49
484.73	6.3114	16.795	393.6	367.23
515.82	6.524	17.7	389.12	360.69
543.76	6.7367	18.604	383.94	354.85
568.77	6.9493	19.509	377.72	349.63
591.17	7.1619	20.413	369.68	344.96
611.31	7.3746	21.318	357.28	340.75
629.52	7.5872	22.222	303.52	336.94
646.07	7.7999	23.127	289.34	333.46
661.22	8.0125	24.031	283.9	330.26
675.15	8.2251	24.936	280.23	327.31
688.03	8.4378	25.84	277.38	324.57
700	8.6504	26.745	275.02	322.02

If the Stirling engine is utilizing an ideal-gas, then inherently ${q_{23}}=-{q_{41}}$; with a real fluid ${q_{23}}>-{q_{41}}$, and thus these values need to be included in Eq. (8).

The efficiency $\eta$ calculated with the values from NIST^54^, derived with Eq. (7), is 13.92%,

$$\begin{aligned} {{\eta }_{NIST}}=-{\frac{{w_{12}}+{w_{34}}}{{q_{23}}+{q_{34}}+{q_{41}}}}=-{\frac{{69,149}-{105,515}}{{47,000}+{252,155}-{37,850}}}={\frac{36,367}{261,305}}=13.92\%. \end{aligned}$$

The efficiency calculated with Eq. (4) is 16.89%,

$$\begin{aligned} {{\eta }_{calc}}=-{\frac{{w_{12}}+{w_{34}}}{{q_{23}}+{q_{34}}+{q_{41}}}}=-{\frac{{69,149}-{105,515}}{{37,603}+{211,232}-{33,516}}}={\frac{36,367}{215,319}}=16.89\%, \end{aligned}$$

which actually exceeds the Carnot efficiency $\eta _C$ of 14.08%!

If one calculates the change in entropy ${\delta }s$ (J/kg${\cdot}{^{\circ }}$C) to the ambient universe with Eq. (6) throughout the full cycle

$$\begin{aligned} {{\delta }s_{NIST}}={{\oint }{\frac{{\delta }q}{T}}}= \left ({\frac{{q_{23}}+{q_{34}}+{q_{41}}}{T_H}}+{\frac{q_{12}}{T_L}}\right)= \left({\frac{261,305}{82+273.15}}+{\frac{-224,939}{32+273.15}}\right)=-1.38,\\ {{\delta }s_{calc}}={{\oint }{\frac{{\delta }q}{T}}}=\left ({\frac{{q_{23}}+{q_{34}}+{q_{41}}}{T_H}}+{\frac{q_{12}}{T_L}}\right)=\left({\frac{215,319}{82+273.15}}+{\frac{-178,953}{32+273.15}}\right)=19.83, \end{aligned}$$

it is noticed that the net total change in entropy per cycle with the NIST^54^ internal energies ${{\delta }s_{NIST}}$ (J/kg${\cdot }{^{\circ }}$C), derived with Eq. (7), obeys Clausius’ Eq. (5); Eq. (7) was in fact originally derived to ensure a thermodynamic cycle obeys Clausius’ Eq. (5). If one assumes the internal energy of a real fluid can be determined with Eq. (4), an empirical equation based on previous measurements of the enthalpy of vaporization^1,25–50^ then the net total change in entropy per cycle ${{\delta }s_{calc}}$ (J/kg${\cdot}{^{\circ }}$C) fails to obeys Clausius’ Eq. (5).

If the theoretical macroscopic Stirling cycle heat engine utilizing real fluids described is to exceed the Carnot efficiency, then Eq. (3) must be the most accurate description of the change in internal energy for a non-ideal fluid. If Clausius’ (Eq. 5) remains applicable in the presence of intermolecular Van der Waals force, however, then Eq. (7) would apply; inherently Eq. (7) would have greater changes in internal potential energy during isothermal compression and expansion of a real fluid (observed in Table 1), and ensuring the ideal Stirling efficiency $\eta$ is less than or equal to the Carnot efficiency ${\eta }_C$ defined in Eq. (1). It is thus desired to perform an experiment to determine which, Eq. (3) or Eq. (7), is the most accurate definition of the change in specific internal energy ${\delta }{u}$ (J/kg) of a non-ideal fluid.

## Experimental description

**Figure 1:**
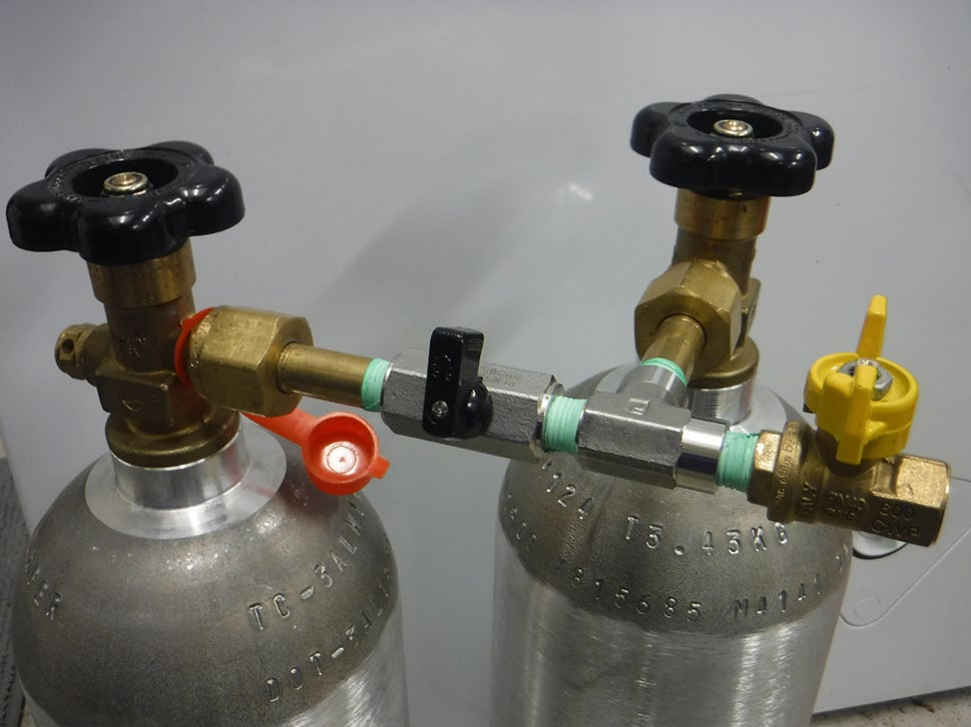
The manifold to connect the two 3.4 liter CO$_2$ cylinders.

To determine if Eq. (3) or Eq. (7) is the most accurate definition of ${\delta }{u}$ (J/kg), a simple and easily repeatable experiment was performed. Two compressed gas cylinders, manufactured by *Luxfur*, with a volume of 3.4 liters each, and designed to hold 5 lbs of carbon dioxide (CO$_2$), were obtained. They were connected by a manifold assembly (Fig. 1) which included adapters from the CGA-320 valve to NPT, a ball valve, a tee, and a separate ball valve (to bleed the small amount of CO$_2$ during disassembly). Three calibrated thermocouples (Taylor # 9940, Panel-Mount LCD Thermometer with Remote Probe; range $-40$ to 150 $^{\circ }$C) were used, with one attached via aluminum tape to each cylinder, and the third attached to the manifold.

Before the experiment could commence, it was necessary to characterize the mass heat capacity $mC_P$ (J/$^{\circ }$C) of both the individual cylinders, as well as the manifold assembly. First, the cylinder or manifold was left in a freezer (maintained at a temperature of $-20\;{^{\circ }}$C) for at least 24 hours, and the temperature inside the freezer was first measured and recorded as $T_{0,Sample}$ ($^{\circ }$C). A mass (kg) of water was first weighed, and this water was poured into a bag resting inside an insulated chest. A thermocouple was left in the bottom of the bag, and the temperature was then collected as the initial temperature of the water $T_{0,Water}$ ($^{\circ }$C). The sample, either the cylinder or the manifold, was quickly moved into the water-filled bag, and insulating material was then piled on top of the bag to the limit that the insulated chest could be securely closed. The water temperature, measured by the thermocouple, would quickly drop and later settle. It was observed that the temperature would often settle after 15 minutes, but the final temperature $T_F$ ($^{\circ }$C) was collected after 60 minutes (minimal difference between the 15 minute measurements). By using the NIST Chemistry WebBook^54^ to determine the heat $Q_{water}$ (J) out of the mass of water from the change in temperature, the heat into the sample could be determined, and the mass heat capacity $mC_P$ (J/$^{\circ }$C) could be estimated

9 $$\begin{aligned} {mC_P} & = {} {\frac{Q_{water}}{{T_F} - {T_{0,Sample}}}}. \end{aligned}$$

This was performed twice with both the cylinder and the manifold, and the results are tabulated in Table 3. The averaged measured mass heat capacity of the cylinder is 2,524 J/${^{\circ }}$C, and the mass heat capacity of the manifold is 454 J/${^{\circ }}$C.

**Table 3:** The results of the effort to find the mass heat capacity $mC_P$ (J/$^{\circ }$C) of the cylinder and the manifold.

Sample	Mass water	$T_0$ *Sample*	$T_0$ *Water*	$T_F$	$mC_P$
	(kg)	($^{\circ }$C)	($^{\circ }$C)	($^{\circ }$C)	(J/$^{\circ }$C)
Cylinder	4.01	$-16.1$	23.7	18.4	2575.23
Cylinder	3.9	$-16.3$	23.2	18	2473.09
Manifold	1.6	$-20$	24.7	21.8	464.56
Manifold	1.77	$-19.3$	24.9	22.4	443.96

**Figure 2:**
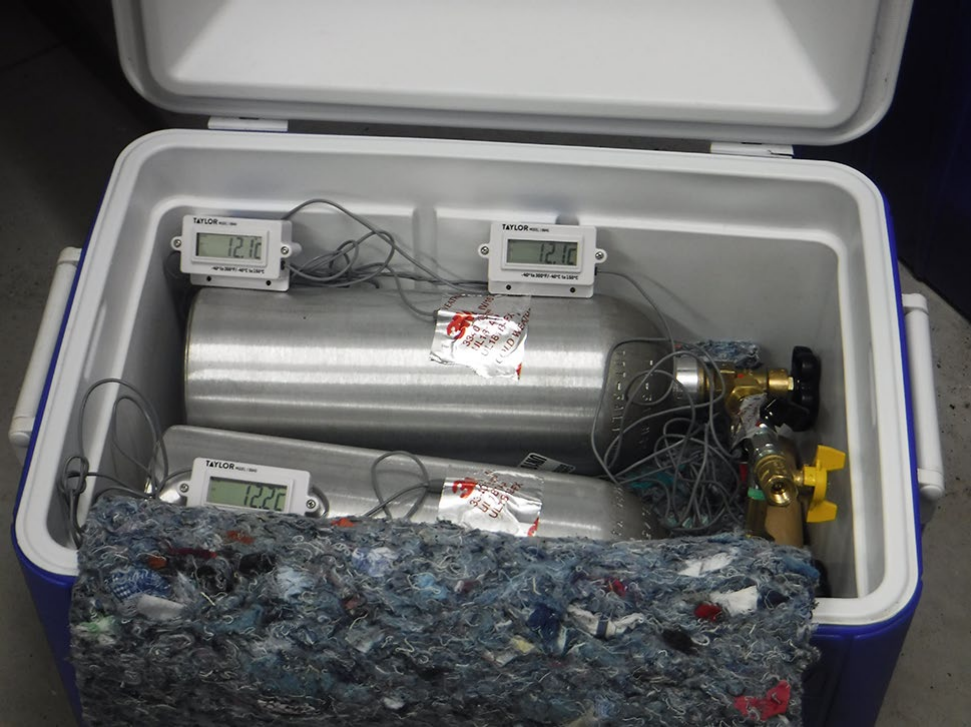
The full experimental apparatus, with two CO$_2$ cylinders connected by the manifold (Fig. 1), inside the insulated chest, with the three thermocouples attached.

The process of the experiment was to have a mass of CO$_2$ in Cylinder 1, and leave Cylinder 2 empty. The mass of CO$_2$ was determined by simply weighing Cylinder 1 before the experiment, and subtracting the measured mass of the empty cylinder (3,460.6 g). The initial temperature on Cylinder 1, Cylinder 2, and the manifold (3) was recorded, and then the two-cylinder assembly was added to the insulated chest and thoroughly covered in insulation (Fig. 2). To commence the experiment, the ball valve was suddenly opened, allowing for CO$_2$ to flow from Cylinder 1 to Cylinder 2, thus instantly doubling the volume and suddenly dropping the temperature due to the Joule–Thomson effect^55^. Over time, the temperature of the cylinders and manifold would drop, as heat would flow from the aluminum cylinders and steel manifold into the cooler CO$_2$ until they reached thermal equilibrium, and the final temperature on Cylinder 1, Cylinder 2, and the manifold (3) was recorded. The results of these temperature measurements are tabulated in Table 4.

**Table 4:** The measured temperature ($^{\circ }$C) of Cylinder *1* and Cylinder *2*, as well as the manifold temperature *3*, both before *0* and after *F* the manifold valve was opened.

Test	$T_{1,0}$	$T_{2,0}$	$T_{3,0}$	$T_{1,F}$	$T_{2,F}$	$T_{F,3}$
Number	($^{\circ }$C)	($^{\circ }$C)	($^{\circ }$C)	($^{\circ }$C)	($^{\circ }$C)	($^{\circ }$C)
1-1	18.4	19.1	19.4	10.7	11.7	13.1
1-2	18.2	18.7	18.3	7.9	19.9	11.8
1-3	17.9	19.3	19.9	6.9	20.3	12.9
1-4	18.4	19.2	18.7	8.4	20.3	12.7
1-5	17.9	18.1	18.6	13.6	19.3	12.4
2-1	15.1	15.6	15.6	7.5	8.2	9.8
2-2	14.7	15.2	16.0	4.8	16.1	8.9
2-3	10.6	14.5	12.9	1.1	15.6	6.9
2-4	14.5	15.5	16.2	3.4	16.7	8.3
2-5	13.5	15.3	16.3	10.2	16.3	12.1
2-6	13.1	13.3	13.2	11.4	14.2	11.6
3-1	10.9	10.0	10.3	3.1	6.6	4.9
3-2	10.2	9.0	9.7	0.7	10.5	4.2
3-3	7.6	8.9	10.2	$-1.7$	10.2	3.8
3-4	4.7	9.4	8.5	$-4.5$	10.4	1.7
3-5	11.2	11.5	11.4	0.4	12.7	5.2
3-6	7.2	11.4	9.9	2.2	11.7	4.1
3-7	8.2	11.2	11.0	7.0	12.0	8.6
4-1	10.9	11.8	12.2	4.5	4.8	6.4
4-2	8.0	9.0	9.2	0.8	5.3	3.9
4-3	9.8	11.1	11.0	0.5	11.6	5.1
4-4	9.4	11.2	10.4	$-0.1$	12.2	4.9
4-5	8.0	11.2	10.9	$-1.6$	12.0	3.8
4-6	11.3	11.8	12.3	6.2	12.6	8.5
4-7	11.6	12.1	12.2	9.9	13.0	10.6

Afterwards, the remaining mass of CO$_2$ in Cylinder 1 and Cylinder 2 was recorded, and these results, along with the initial mass in Cylinder 1, are tabulated in Table 5. In addition, with the known temperatures (Table 4) and densities (mass over the 3.4 liter volume), the specific internal energy *u* (kJ/kg) values as determined by the NIST Chemistry WebBook^54^ (which were derived by NIST with Eq. (7)) were collected and tabulated in Table 5. On average, less than 1% of the CO$_2$ was lost in the disassembly of the manifold or due to leaking. In addition, it was frequently observed that only a small portion of the mass (approximately 400 g) would travel from Cylinder 1 to Cylinder 2; this is expected, as much of the liquid CO$_2$ (and thus most of the mass) would stay in the original Cylinder 1 rather than travel through the manifold. It was also noticed that at times Cylinder 2 would experience a slight *increase* in temperature; mainly due to the kinetic energy of expansion, and the low masses of CO$_2$ that made its way into Cylinder 2.

**Table 5:** The measured mass (g) of CO$_2$ in the 3.4 liter cylinders, as well as the specific internal energy *u* (kJ/kg) collected from the NIST webbook^54^.

Test	$mass_0$	$mass_{F1}$	$mass_{F2}$	$u_{0}$	$u_{F1}$	$u_{F1}$
Number	(g)	(g)	(g)	(kJ/kg)	(kJ/kg)	(kJ/kg)
1-1	2026.7	1565.6	459.8	256.95	249.92	391.15
1-2	1565.2	1200.2	364.1	272.42	258.99	408.49
1-3	1199.1	848.4	350.8	292.83	285.54	410.19
1-4	847.4	484.4	342.4	332.90	370.17	411.06
1-5	483.8	246.9	236.3	394.34	415.90	421.39
2-1	1880.4	1385.4	491.0	251.91	247.99	366.59
2-2	1385.4	1054.0	332.0	270.20	258.64	408.73
2-3	1053.8	764.2	288.0	278.59	273.67	413.02
2-4	764.0	444.7	305.1	329.22	357.58	412.07
2-5	444.6	225.9	216.4	394.46	415.61	421.24
2-6	226.3	110.9	109.5	417.76	258.16	431.34
3-1	1941.2	1582.5	349.8	238.88	227.90	398.84
3-2	1582.2	1270.0	299.9	247.85	233.03	407.66
3-3	1270.0	995.4	272.2	254.02	241.40	410.48
3-4	995.8	738.6	238.5	262.85	255.59	414.36
3-5	735.7	442.4	283.4	319.53	341.88	411.23
3-6	442.6	197.7	182.7	380.36	412.78	421.50
3-7	197.6	97.2	94.8	417.26	427.74	431.45
4-1	2233.5	1679.9	551.5	232.55	228.77	329.70
4-2	1679.9	1356.8	321.0	238.32	229.54	401.02
4-3	1356.8	1058.5	296.0	256.33	244.19	408.97
4-4	1058.4	773.8	283.2	274.00	267.86	410.85
4-5	773.5	503.8	268.1	299.86	311.51	412.35
4-6	503.6	257.5	244.1	379.36	408.98	415.44
4-7	257.2	128.7	126.6	413.23	426.21	428.62

## Experimental analysis

Carbon dioxide (CO$_2$)^56^ has a critical temperature $T_C$ of 304.128 K; a critical pressure $P_C$ of 7,377,300 Pa; a critical specific density $\rho _C$ of 467.6 (kg/m$^3$), 3 degrees of freedom, a molar mass of 44 g/mole, and a Pitzer eccentric factor^57^ of 0.228. The specific gas constant $R_G$ for CO$_2$ is 188.924 J/kg${\cdot}{^{\circ }}$C; the ideal-gas specific heat at a constant volume $C_V=3.5{\cdot }{R_G}$; and the specific heat ratio ${\kappa }={\frac{C_P}{C_V}}={\frac{4.5}{3.5}}=1.28$. For CO$_2$, the value of *a’* as defined in Eq. (4) is 728.46 Pa K$^{0.25}$ m$^6$/kg$^{2}$.

The density of saturated liquid CO$_2$ $\rho _L$ (kg/m$^3$) and saturated gas CO$_2$ $\rho _G$ (kg/m$^3$) is defined with Eq. (10)^56^

10 $$\begin{aligned} {ln\left(\frac{\rho _L}{\rho _C}\right)} & = {} {{\Sigma }_{i=1}^4}{a_i}{\cdot }{\left(1-{\frac{T}{T_C}}\right)^{t_i}}, \nonumber \\ {ln\left(\frac{\rho _G}{\rho _C}\right)} & = {} {{\Sigma }_{i=1}^5}{b_i}{\cdot }{\left(1-{\frac{T}{T_C}}\right)^{u_i}}, \end{aligned}$$

where $T_C$ is 304.128 K, $\rho _C$ is 467.6 (kg/m$^3$), and the values of $a_i$, $t_i$, $b_i$, and $u_i$ are tabulated in Table 6.

**Table 6:** Coefficient values for Eq. (10).

	1	2	3	4	5
$a_i$	1.9245108	$-0.62385555$	$-0.32731127$	0.39245142	–
$t_i$	0.340	0.5	(10/6)	(11.6)	–
$b_i$	$-1.7074879$	$-0.8227467$	$-4.6008549$	$-10.111178$	$-29.742252$
$u_i$	0.340	0.5	1	(7/3)	(14/3)

Table 7 contains the tabulated densities $\rho$ (kg/m$^3$) of the CO$_2$, both before the experiment $\rho _0$ (kg/m$^3$), and after the experiment in Cylinder 1 $\rho _{F1}$ (kg/m$^3$) and Cylinder 2 $\rho _{F2}$ (kg/m$^3$). In addition, the densities of a saturated liquid $\rho _L$ (kg/m$^3$) and a saturated gas $\rho _G$ (kg/m$^3$) for the experimentally measured CO$_2$ temperatures (Table 4) as determined with Eq. (10) is also tabulated in Table 7. With the density $\rho$ (kg/m$^3$), saturated liquid density $\rho _L$ (kg/m$^3$), and saturated gas density $\rho _G$ (kg/m$^3$), the vapor quality *X* was determined with Eq. (11), and tabulated in Table 8.

11 $$\begin{aligned} X&=&\frac{(1/{\rho})-(1/{\rho_L})}{(1/{\rho_G})-(1/{\rho_L})}=\frac{{v}-{v_L}}{{v_G}-{v_L}} \end{aligned}$$

**Table 7:** The calculated density of CO$_2$ in the 3.4 liter cylinders, taken from the mass tabulated in Table 5. The densities of a saturated liquid and a saturated gas are defined with Eq. (10).

Test	$\rho _{0}$	$\rho _{0-L}$	$\rho _{0-G}$	$\rho _{F1}$	$\rho _{F1-L}$	$\rho _{F1-G}$	$\rho _{F2}$	$\rho _{F2-L}$	$\rho _{F2-G}$
Num	($\frac{kg}{m^3}$)	($\frac{kg}{m^3}$)	($\frac{kg}{m^3}$)	($\frac{kg}{m^3}$)	($\frac{kg}{m^3}$)	($\frac{kg}{m^3}$)	($\frac{kg}{m^3}$)	($\frac{kg}{m^3}$)	($\frac{kg}{m^3}$)
1-1	596.09	789.83	182.35	460.47	855.86	138.39	135.22	848.19	143.19
1-2	460.35	791.80	180.96	353.00	876.26	126.02	107.09	774.46	193.42
1-3	352.68	794.72	178.89	249.53	883.22	121.94	103.18	770.16	196.58
1-4	249.24	789.83	182.35	142.47	872.72	128.12	100.71	770.16	196.58
1-5	142.29	794.72	178.89	72.62	832.99	152.94	69.50	780.74	188.86
2-1	553.06	820.32	161.31	407.47	879.06	124.37	144.41	874.14	127.28
2-2	407.47	823.76	159.02	310.00	897.32	113.88	97.65	811.50	167.27
2-3	309.94	856.61	137.92	224.76	920.75	101.12	84.71	815.95	164.25
2-4	224.71	825.47	157.89	130.79	906.39	108.85	89.74	806.04	171.01
2-5	130.76	833.81	152.40	66.44	859.61	136.07	63.65	809.69	168.51
2-6	66.56	837.08	150.29	32.62	850.51	141.72	32.21	828.00	156.21
3-1	570.94	854.34	139.33	465.44	908.30	107.80	102.88	885.27	120.75
3-2	465.35	859.61	136.07	373.53	923.18	99.84	88.21	857.36	137.46
3-3	373.53	878.37	124.78	292.76	937.45	92.52	80.06	859.61	136.07
3-4	292.88	897.98	113.51	217.24	953.40	84.68	70.15	858.11	136.99
3-5	216.38	852.05	140.76	130.12	925.00	98.89	83.35	840.30	148.21
3-6	130.18	881.15	123.15	58.15	913.96	104.74	53.74	848.19	143.19
3-7	58.12	874.14	127.28	28.59	882.53	122.34	27.88	845.85	144.67
4-1	656.91	854.34	139.33	494.09	899.29	112.78	162.21	897.32	113.88
4-2	494.09	875.56	126.44	399.06	922.58	100.16	94.41	894.02	115.74
4-3	399.06	862.58	134.25	311.32	924.40	99.21	87.06	848.97	142.70
4-4	311.29	865.51	132.46	227.59	928.01	97.33	83.29	844.28	145.67
4-5	227.50	875.56	126.44	148.18	936.87	92.81	78.85	845.85	144.67
4-6	148.12	851.28	141.24	75.74	887.99	119.18	71.79	841.10	147.70
4-7	75.65	848.97	142.70	37.85	861.84	134.70	37.24	837.89	149.76

**Table 8:** The calculated vapor quality solved with Eq. (11), utilizing the density $\rho$ (kg/m$^3$), saturated liquid density $\rho _L$ (kg/m$^3$), and saturated gas density $\rho _G$ (kg/m$^3$) tabulated in Table 7.

Test	$X_{0}$	$X_{F1}$	$X_{F2}$
1-1	0.0976	0.1656	1.0709
1-2	0.2133	0.2490	2.0746
1-3	0.3641	0.4068	2.2155
1-4	0.6511	0.8820	2.2782
1-5	1.3319	2.3548	3.2654
2-1	0.1183	0.1907	0.8611
2-2	0.2444	0.2754	1.8982
2-3	0.3385	0.3820	2.1758
2-4	0.6323	0.8093	2.1497
2-5	1.2025	2.2451	3.0805
2-6	2.5332	5.0139	5.7458
3-1	0.0967	0.1281	1.2011
3-2	0.1593	0.1784	1.6650
3-3	0.2238	0.2411	1.8312
3-4	0.2989	0.3303	2.1340
3-5	0.5814	0.7313	1.9447
3-6	0.9372	1.9050	3.0028
3-7	2.3928	4.8072	6.0527
4-1	0.0586	0.1176	0.6588
4-2	0.1303	0.1598	1.2595
4-3	0.2141	0.2368	1.7682
4-4	0.3217	0.3606	1.9050
4-5	0.4808	0.5853	2.0069
4-6	0.9443	1.6626	2.2824
4-7	2.0654	4.0326	4.6798

Utilizing the density $\rho$ (kg/m$^3$), saturated liquid density $\rho _L$ (kg/m$^3$), and saturated gas density $\rho _G$ (kg/m$^3$) tabulated in Table 7, as well as the experimentally measured temperatures tabulated in Table 4, and the masses of CO$_2$ tabulated in Table 5, the internal energy *U* (kJ) was calculated using the empirical Eq. (4), and tabulated in Table 9.

**Table 9:** The calculated internal energy *U* (kJ), solved with Eq. (4), utilizing the measured temperature in Table 4, and the densities tabulated in Table 7.

Test	$U_{0x}$	$U_{0-L}$	$U_{0-G}$	$U_{F1-x}$	$U_{F1-L}$	$U_{F1-G}$	$U_{F2-x}$	$U_{F2-L}$	$U_{F2-G}$
Num	(kJ)	(kJ)	(kJ)	(kJ)	(kJ)	(kJ)	(kJ)	(kJ)	(kJ)
1-1	177.74	108.52	325.56	165.91	56.05	255.40	75.57	17.45	74.92
1-2	174.49	83.02	251.60	147.67	35.93	196.14	63.69	20.91	58.15
1-3	156.19	62.70	192.94	119.41	23.67	138.68	61.70	20.52	55.93
1-4	126.13	45.37	136.12	77.91	15.00	79.14	60.37	20.03	54.59
1-5	80.97	25.30	77.84	43.64	10.41	40.13	42.80	13.20	37.83
2-1	174.55	85.70	304.78	156.63	40.34	226.43	78.73	15.00	80.23
2-2	163.86	61.86	224.73	135.42	24.98	172.30	57.77	15.91	53.69
2-3	139.75	37.50	171.92	107.84	12.63	124.75	50.68	13.46	46.63
2-4	114.95	33.76	123.98	70.93	9.32	72.67	53.64	15.06	49.26
2-5	73.98	18.64	72.27	39.66	7.85	36.87	38.99	10.47	34.98
2-6	40.17	9.29	36.81	20.22	4.14	18.08	20.18	4.76	17.78
3-1	167.94	70.32	316.61	157.46	32.23	258.59	58.30	9.55	57.18
3-2	165.71	54.96	258.22	145.02	20.02	207.26	51.55	10.61	48.93
3-3	151.34	37.24	207.56	126.37	11.20	162.14	47.13	9.45	44.42
3-4	130.91	23.40	162.78	102.34	4.50	119.95	41.75	8.39	38.92
3-5	110.09	27.13	119.96	69.71	6.72	72.18	49.38	11.38	46.13
3-6	71.79	12.62	72.34	33.94	3.68	32.29	32.67	6.93	29.77
3-7	34.72	6.04	32.29	17.51	2.73	15.89	17.41	3.66	15.44
4-1	159.16	80.91	364.28	160.29	38.82	274.61	85.40	13.07	90.16
4-2	164.64	50.64	274.52	148.83	21.64	221.44	53.70	7.93	52.48
4-3	157.68	45.98	221.50	132.51	16.28	172.72	51.16	11.17	48.24
4-4	139.20	34.98	172.83	108.15	11.02	126.21	49.25	11.06	46.12
4-5	112.49	23.32	126.40	77.07	5.76	82.07	46.80	10.35	43.67
4-6	81.49	18.68	82.10	44.09	6.82	42.10	43.02	9.75	39.73
4-7	44.98	9.71	41.92	23.22	4.39	21.01	23.12	5.17	20.60

For qualities *X* greater than 1, the CO$_2$ is treated as a vapor, and the internal energy is estimated solely with the empirical Eq. (4), and the final internal energy *U* (kJ) was tabulated in Table 10. For qualities *X* less than 1 (there were no measurements at a greater density than the saturated liquid density), the internal energy of the liquid–vapor mixture *U* (kJ) was calculated with Eq. (12) from the quality *X* (Eq. (11)), saturated liquid internal energy $U_L$ (kJ), and the saturated gas internal energy $U_G$ (kJ), both tabulated in Table 9.

12 $$\begin{aligned} U &= {} {{U_L}{\cdot }{(1-X)}}+{{U_G}{\cdot }{X}}. \end{aligned}$$

**Table 10:** The calculated internal energies *U* (kJ), solved with Eq. (4), adjusting for mixed liquid–vapor, using the internals energies *U* (kJ) tabulated in Table 9, with the qualities *X* tabulated in Table 8, solved with Eq. (12).

Test	$U_{0}$	$U_{F1}$	$U_{F2}$
Num	(kJ)	(kJ)	(kJ)
1-1	129.69	89.06	75.57
1-2	118.97	75.82	63.69
1-3	110.12	70.46	61.70
1-4	104.46	71.57	60.37
1-5	80.97	43.64	42.80
2-1	111.61	75.84	71.17
2-2	101.66	65.55	57.77
2-3	83.00	55.46	50.68
2-4	90.81	60.59	53.64
2-5	73.98	39.66	38.99
2-6	40.17	20.22	20.18
3-1	94.15	61.24	58.30
3-2	87.34	53.43	51.55
3-3	75.36	47.59	47.13
3-4	65.07	42.64	41.75
3-5	81.09	54.60	49.38
3-6	68.60	33.94	32.67
3-7	34.72	17.51	17.41
4-1	97.51	66.55	63.85
4-2	79.81	53.57	53.70
4-3	83.56	53.32	51.16
4-4	79.33	52.56	49.25
4-5	72.88	50.42	46.80
4-6	78.57	44.09	43.02
4-7	44.98	23.22	23.12

**Table 11:** The combined energy input *Q* (kJ) into the CO$_2$, defined with Eqs. (13) and (14), using the theory defined in Eq. (4), from NIST in Table 5^54^, and measured experimentally. The parentheses represent the percent (%) error with $Q_{EXP}$. These tabulated results are plotted in Figure 3.

Test	$Q_{Theory}$	$Q_{NIST}$	$Q_{EXP}$
Num	(kJ)	(kJ)	(kJ)
1-1	34.9442 (14.71%)	50.3454 (22.88%)	40.9726
1-2	20.5379 (20.76%)	33.1792 (28.01%)	25.9194
1-3	22.0339 (22.47%)	35.0143 (23.21%)	28.4180
1-4	27.4844 (9.12%)	37.9578 (50.70%)	25.1876
1-5	5.4758 (48.53%)	11.4785 (7.89%)	10.6392
2-1	35.3962 (12.59%)	49.8695 (23.16%)	40.4932
2-2	21.6599 (16.50%)	33.9698 (30.96%)	25.9394
2-3	23.1390 (3.29%)	34.5102 (44.24%)	23.9256
2-4	23.4268 (18.01%)	33.2143 (16.24%)	28.5742
2-5	4.6671 (39.48%)	9.6657 (25.33%)	7.7120
2-6	0.2402 (91.25%)	$-18.6774$ (780.27%)	2.7456
3-1	25.3884 (17.36%)	36.4521 (18.66%)	30.7204
3-2	17.6409 (22.25%)	26.0571 (14.84%)	22.6890
3-3	19.3648 (16.16%)	29.4168 (27.36%)	23.0976
3-4	19.3141 (18.79%)	25.8576 (8.72%)	23.7840
3-5	22.8826 (15.39%)	32.7121 (20.95%)	27.0452
3-6	$-1.9849$ (113.69%)	$-9.7327$ (167.14%)	14.4960
3-7	0.1985 (90.54%)	0.0272 (98.70%)	2.0992
4-1	32.8876 (9.79%)	46.7398 (28.21%)	36.4548
4-2	27.4498 (8.25%)	39.8135 (33.08%)	29.9178
4-3	20.9232 (15.94%)	31.7417 (27.53%)	24.8898
4-4	22.4886 (6.11%)	33.6212 (40.37%)	23.9510
4-5	24.3473 (4.28%)	35.5481 (39.76%)	25.4346
4-6	8.5325 (32.17%)	15.6756 (24.62%)	12.5784
4-7	1.3649 (50.29%)	2.8338 (3.21%)	2.7456

All of these final internal energies *U* (kJ) are tabulated in Table 10.

**Figure 3:**
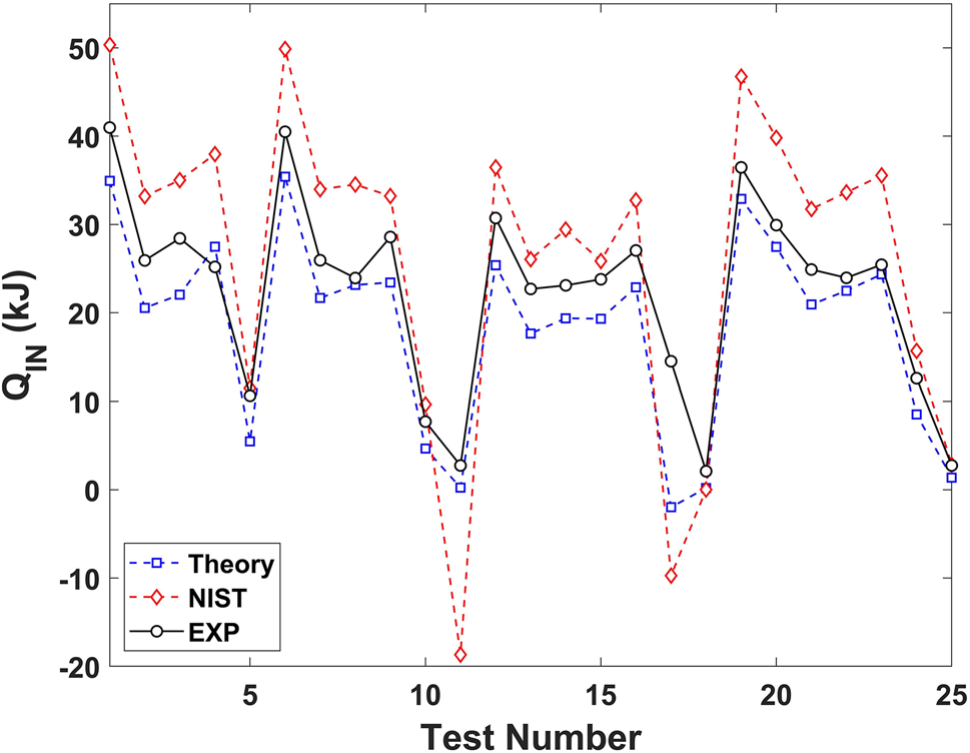
The combined energy input *Q* (kJ) into the CO$_2$, defined with Eqs. (13) and (14), using the theory defined in Eq. (4), from NIST in Table 5^54^, and measured experimentally. These plotted results are tabulated in Table 11.

Finally, the estimated heat inputs were determined with Eq. (13), plotted in Figure 3, and tabulated in Table 11. These include the heat estimates $Q_{theory}$ (kJ) utilizing the internal energies derived from the empirical Eq. (4) and tabulated in Table 10; as well as the heat estimates $Q_{NIST}$ (kJ) utilizing the specific internal energies collected from the NIST Chemistry WebBook^54^ and tabulated in Table 5.

13 $$\begin{aligned} Q &= {} {U_{F1}}+{U_{F2}}-{U_0}. \end{aligned}$$

The values of $Q_{theory}$ (kJ) and $Q_{NIST}$ (kJ) are compared to the experimentally measured heat inputs $Q_{EXP}$ (kJ), determined by comparing the measured changes in temperature (Table 4) with the mass heat capacity tabulated in Table 3, as described in Eq. (14),

14 $$\begin{aligned} {Q_{EXP}}={{{mC_{P,Cylinder}}{\cdot }{({T_{1,0}}+{T_{2,0}}-{T_{1,F}}-{T_{2,F}})}}+{{mC_{P,Manifold}}{\cdot }{({T_{3,0}}-{T_{3,F}})}}}. \end{aligned}$$

## Conclusion

When analyzing the results of Table 11 and Fig. 3, with 25 independent test results, the correlation between $Q_{Theory}$ (Eq. 3) and $Q_{EXP}$ is 0.9560; in excess of the correlation of 0.9229 between $Q_{NIST}$ (Eq. 7) and $Q_{EXP}$. The average error between $Q_{Theory}$ and $Q_{EXP}$ is 29%, less than the average error of 64% between $Q_{NIST}$ and $Q_{EXP}$. The median error between $Q_{Theory}$ and $Q_{EXP}$ is 17%, less than the median error of 27% between $Q_{NIST}$ and $Q_{EXP}$. Finally, the standard deviation of the error between $Q_{Theory}$ and $Q_{EXP}$ is 29%, less than the standard deviation of the error of 153% between $Q_{NIST}$ and $Q_{EXP}$. The experimental data suggests that Eq. (3) is the most accurate definition of the change in internal energy of a real fluid ${\delta }{u}$ (J/kg), as compared to Eq. (7). This effort provides an experimental justification to the possibility of the theoretical macroscopic Stirling cycle heat engine utilizing real fluids described earlier^1^ exceeding the Carnot efficiency.

## Data Availability

All data generated or analysed during this study are included in this published article [and its supplementary information files].
